# Hormones and Hemodynamics in Pregnancy

**DOI:** 10.5812/ijem.14098

**Published:** 2014-04-01

**Authors:** Oleksandra Tkachenko, Dmitry Shchekochikhin, Robert W. Schrier

**Affiliations:** 1 Division of Renal Diseases and Hypertension, University of Colorado Denver, Denver, Colorado, USA

**Keywords:** Pregnancy, Arterial, Osmolar Concentration, Relaxin, Nitric Oxide, Pre-Eclampsia, Endothelium

## Abstract

**Context:**

Normal pregnancy is associated with sodium and water retention, which results in plasma volume expansion prior to placental implantation. The explanation offered for these events is that pregnancy ‘resets’ both volume and osmoreceptors.

**Evidence Acquisition:**

The mechanisms for such an enigmatic ‘resetting’ in pregnancy have not previously been explained. However, recent human pregnancy studies have demonstrated that the earliest hemodynamic change in pregnancy is primary systemic arterial vasodilation. This arterial underfilling is associated with a secondary increase in cardiac output and activation of the neurohumoral axis, including stimulation of the renin-angiotensin-aldosterone, sympathetic, and non-osmotic vasopressin systems. Resistance to the pressor effects of angiotensin and sympathetic stimulation in pregnancy is compatible with an increase in endothelial nitric oxide synthase activity.

**Results:**

In contrast to the sodium and water retention which occur secondary to the primary arterial vasodilation in cirrhosis, glomerular filtration and renal blood flow are significantly increased in normal pregnancy. A possible explanation for this difference in arterial vasodilation states is that relaxin, an arterial vasodilator which increases during pregnancy, has a potent effect on both systemic and renal circulation. Endothelial damage in pregnancy is pivotal in the pathogenesis of preeclampsia in pregnancy.

**Conclusions:**

Against a background of the primary arterial vasodilation hypothesis, it is obvious that reversal of the systemic vasodilatation in pregnancy, without subsequent activation of the renin-angiotensin-aldosterone system (78), will evoke a reversal of all the links in the chain of events in normal pregnancy adaptation, thus, it may cause preeclampsia. Namely, a decrease of renal vasodilation will decrease glomerular filtration rate.

## 1. Context

Normal human pregnancy is characterized by physiologic changes in neurohumoral status, systemic and renal hemodynamics, as well as changes in sodium and water balance. Specifically, there is a decrease in mean arterial pressure (MAP) (1) and plasma osmolality (1-3) and an increase in total body electrolytes and water (3, 4). Decreased blood pressure occurs in spite of increased activation of the renin-angiotensin-aldosterone system (RAAS) (5, 6). The causes and sequence of all these phenomena have not been completely defined. Nevertheless, primary systemic vasodilatation is a pivotal feature of pregnancy. Studies in animals (7) and humans (1) have shown that hemodynamic changes in pregnancy occur early, prior to complete placentation. These gestational alterations are qualitatively comparable to the luteal phase of the menstrual cycle, but to a lesser degree (8, 9). Similarly, exaggerated changes occur during ovarian hyperstimulation (10). Changes in sodium and water status are the other remarkable adaptations of normal pregnancy in humans. They are characterized by increases in total body water by 8 L (4), and total body sodium by 1000 mEq (11), as plasma osmolality falls (1-3, 12) by 10 mosmol/kg below nonpregnant levels (1, 13).

## 2. Evidence Acquisition

### 2.1. Pregnancy is a unique state of arterial underfilling

**Figure 1.**
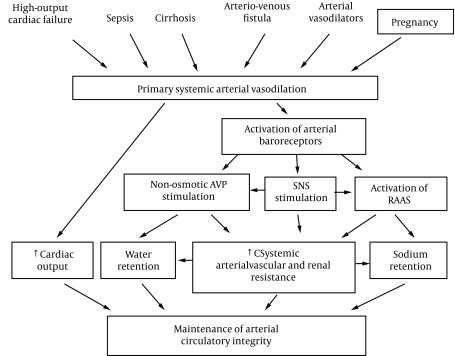
Body Fluid Regulation in Health and Disease: A Unifying Hypothesis., Renin-Angiotensin-Aldosterone System (RAAS); Sympathetic Nervous System (SNS). Reproduced with Permission from Ref. 19.

**Figure 2.**
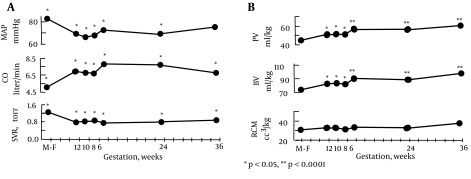
Ten Women Studied in the Mid-Follicular Phase of the Menstrual Cycle and Weeks 6, 8, 10, 12, 24 and 36 Gestation. Reproduced with Permission from Ref. 1. A. Systemic hemodynamic changes throughout early human pregnancy. Mean arterial pressure (MAP) decreased and cardiac output (CO) increased significantly by week 6 gestation in association with a decrease in systemic vascular resistance (SVR). *P < 0.05, **P < 0.001. Figure 2B . Plasma volume (PV), blood volume (BV), and red cell mass (RCM) determinations in early pregnancy. Plasma and blood volume increased significantly by week 6 gestation. Red cell mass remained unchanged throughout pregnancy. *P < 0.05, **P < 0.0001.

**Figure 3.**
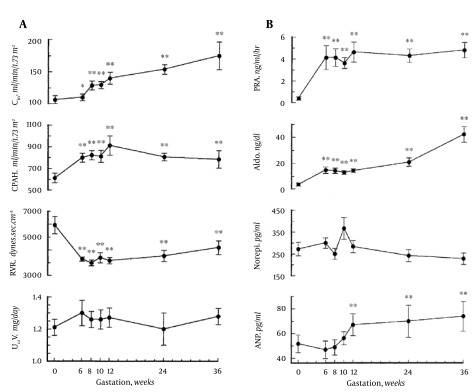
Ten Women Were Studied in the Mid-Follicular Phase of the Menstrual Cycle and Weeks 6, 8, 10, 12, 24 and 36 Gestation Reproduced With Permission From Ref. 19 A. Renal hemodynamic changes throughout early human pregnancy. Renal plasma flow and glomerular filtration rates increased significantly in association with a renal vascular resistance by week 6 gestation. Twenty-four-hour urinary creatinine excretion remained unchanged throughout gestation. Abbreviations: CIn, inulin clearance; CPAH, para-aminohippurate clearance; RVR, renal vascular resistance; UCrV, urinary creatinine excretion. *P < 0.05, **P < 0.001. B. Vasopressor hormone profiles throughout early human pregnancy. Plasma renin activity (PRA) and aldosterone (Aldo) levels increased by week 6 gestation. Norepinephrine (Norepi) concentrations did not change throughout gestation. Atrial natriuretic peptide (ANP) concentrations increased significantly by week 12 gestation (**P < 0.001.).

The primary arterial vasodilation hypothesis of sodium and water retention explains many of these pregnancy changes (14). Thus, the unifying hypothesis of body fluid volume regulation can apply to pregnancy. A normal kidney regulates sodium and water excretion, not primarily in response to total blood volume, but rather by the effective arterial blood volume (EABV) (15). A diminished cardiac output or primary arterial vasodilation triggers a decrease in EABV. This leads to the activation of the RAAS and sympathetic nervous systems and non-osmotic release of arginine vasopressin (AVP), with resultant renal sodium and water retention to compensate for the decrease in EABV. The stretch arterial baroreceptors can be unloaded with stimulation of the neurohumoral axis and cardiac output, even in the presence of increased total blood volume, since an estimated 85% of circulatory blood resides in the venous circulation. The remaining 15% of blood is located in arterial circulation and primarily determines renal sodium and water regulation. Since pregnancy is a unique state of arterial under-filling, its physiology can be understood in the light of other states involving arterial underfilling, such as; hepatorenal or cardiorenal syndromes (Figure 1) (14). However, it should be emphasized that pregnancy has several unique features, namely escape from the sodium-retaining effects of aldosterone, increase in glomerular filtration rate (GFR), and renal blood flow (RBF), in spite of a decrement in systemic vascular resistance (SVR). The initial systemic and renal hemodynamic and neurohumoral changes that occur in early human pregnancy have only now been well defined (Figures 2, 3) (1). SVR decreases significantly by week 6 of gestation and causes a fall in MAP, which leads to a compensatory increase in cardiac output (1, 16). Renal vasodilation occurs simultaneously with systemic vasodilatation and it is also associated with a 30-50% increase in renal blood flow and GFR (1, 17). These increases in renal hemodynamics distinguish pregnancy from other states of systemic vasodilation in which renal vasoconstriction occurs, as a rise in cardiac output is inadequate to maintain MAP. The RAAS is stimulated and circulating levels of renin, angiotensin II (ANG II) and aldosterone rise in early pregnancy (5, 6, 18). This hormonal activation causes sodium and water retention, which leads to the expansion of total plasma volume (1). As might be expected, an increment in atrial natriuretic peptide (ANP) follows volume expansion in pregnancy (1). However an increase in GFR (19) and cardiac output (20) precedes blood volume expansion, and therefore it is related to primary arterial vasodilation of the renal and systemic circulation. Studies of pregnant baboons have also demonstrated that the blood volume expansion occurs after a decrease in SVR and stimulation of the RAAS axis (20). If the blood volume expansion were primary, rather than secondary to the arterial vasodilatation, suppression, not stimulation, of the RAAS would be expected. Unlike other states with primary vasodilatation, escape from the sodium-retaining effects of aldosterone occurs in pregnancy (17).

Besides the increase in progesterone (14), the rise in GFR in pregnancy increases distal sodium delivery, thereby, allowing for further escape from the sodium retaining effect of aldosterone (21). There is also the involvement of the distal tubule in aldosterone sodium escape (21). The volume expansion secondary to aldosterone increases ANP which inhibits sodium reabsorption in the distal tubule. However, angiotensin-mediated upregulation of the epithelial sodium channel (ENaC) and phosphodiesterases in the distal tubule opposes the effect of ANP (22). With respect to the effect of aldosterone to increase potassium secretion in the distal tubule, the increased distal sodium delivery with aldosterone-mediated volume expansion will enhance this effect on potassium excretion (23). A genetic variant of ENaC has been found to be associated with preeclampsia (24).

### 2.2. Volume Expansion

As mentioned previously, water and sodium-retaining hormones are stimulated in pregnancy leading to renal sodium and water retention. Resultant plasma volume expansion compensates for arterial underfilling, which occurs with primary arterial vasodilation. This volume expansion is extremely important for normal fetal development in either animal or human pregnancy. Indeed, water deprivation and salt restriction causes a reduction of fetal weight in rats (25, 26). In humans, idiopathic fetal growth restriction was also associated with reduced plasma volume (27).

### 2.3. Osmoregulation

Another prominent feature of healthy pregnancy is the occurrence of hypoosmolality. This water retention and decreased body tonicity in pregnancy has been a focus of previous investigations (13). Negative free water clearance in healthy pregnant women indicates that free water is steadily retained during pregnancy (28). Rats are an excellent animal model to study osmoregulation in pregnancy, because their blood contains no detectable vasopressinase (29). In humans, vasopressinase is produced by the placenta and it inactivates AVP by clipping the hormone’s first amino acid (30). Thus, the measurement of plasma vasopressin in human pregnancy necessitates the inhibition of vasopressinase (29). An animal study has shown that the threshold for AVP secretion decreases in rat pregnancy, even though urinary diluting and concentrating ability is preserved (31). Studies in human pregnancy have shown comparable results (32, 33). This led to the assumption that in pregnancy, osmoregulation is ‘reset’ at a lower osmolality around a new steady state (31). The threshold for thirst was also found to be ‘reset’ at a lower osmolality plasma level, thereby facilitating water retention (31, 33). The primary arterial vasodilation hypothesis of sodium and water retention in pregnancy is compatible with this ‘reset’ osmostat as mediated by nonosmotic AVP stimulation (14). The finding that plasma AVP is not suppressed in pregnancy in the presence of hyponatremia and hypotonicity supports this hypothesis (31, 33, 34). The urinary excretion of AVP is also increased in pregnancy and relates inversely to urine flow rate and free water clearance. This further supports the role of AVP in hyponatremia in pregnancy (28).

**Figure 4.**
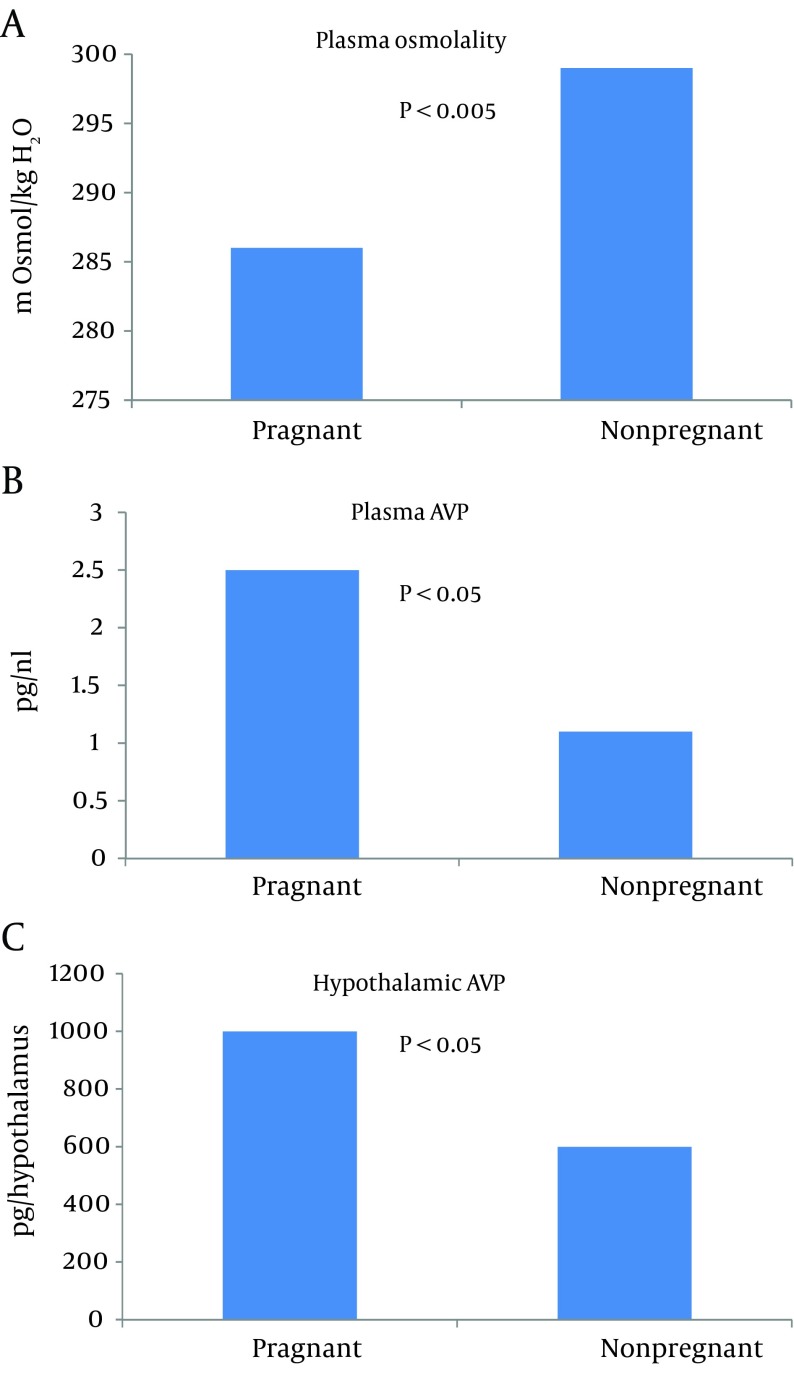
Hypoosmolality in Pregnancy (A) Associated with Increased Plasma Arginine Vasopressin (AVP) (B) and a Rise in Hypothalamic AVP (C).

**Figure 5.**
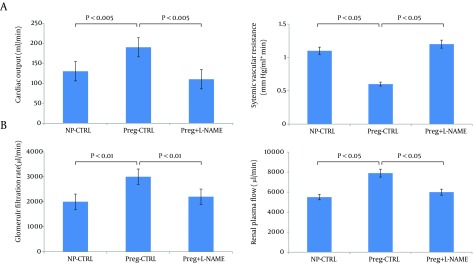
Chronic Nitric Oxide Synthase (NOS) Inhibition Returns Cardiac Output (CO) and Systemic Vascular Resistance (SVR) in Day 14 Pregnant Rats to Nonpregnant Levels. Values are means + /- SE for CO (A), and calculated SVR (A). *P < 0.05 vs. NP-control (CTRL) and vs. pregnant (Preg) + nitro-L-arginine methyl ester (NAME). Chronic NOS inhibition reverses glomerular hyperfiltration and renal vasodilation in day 14 pregnant rats. B: glomerular filtration rate (GFR); renal plasma flow (RPF). *P < 0.01 vs. NP-CTRL and vs. Preg+NAME; **P < 0.05 vs. NP-CTRL. Reproduced with permission from Ref. 50.

Xu et al. were able to show not only increased plasma AVP, but also an increase in hypothalamic AVP mRNA in pregnant rats in the presence of hypoosmolality (Figure 4) (35). Vasopressin-mediated water reabsorption in the collecting duct occurs via aquaporin 2 (AQP2) water channels by the activation of vasopressin 2 receptors (36). Since upregulation of AQP2 was observed in other states of arterial underfilling, namely cirrhosis (37) and cardiac failure (38), it was extremely important to measure AQP2 expression in pregnancy. A significant increase in AQP2 was observed by day 7 of gestation (Figure 5) (34). Administration of V2 receptor antagonist led to diuresis, urinary dilution and decrease in the AQP2 protein to nonpregnant levels. The unexpected persistence of hypoosmolality, despite the V2 receptor blockade, can be explained by polydipsia considering the ‘reset’ of the thirst threshold. This confirmed the role of V2 receptors in the upregulation of AQP2 during pregnancy. Similar results were demonstrated in human pregnancy (39).

### 2.4. Potential Mediation of Vasodilation of Pregnancy

Relaxin, the peptide hormone produced by the corpus luteum under the stimulation of human chorionic gonadotropin (HCG), has been suggested to play an important role in the regulation of the hemodynamics and water metabolism in pregnancy. Chronic administration of a recombinant human relaxin (rhRLX) to nonpregnant animals increased cardiac output and arterial compliance (40), reduced SVR (40, 41), and decreased renal vascular resistance (RVR) (42), in association with increased RBF and GFR. In human studies, acute infusion of rhRLX increased the cardiac index, and reduced SVR, serum creatinine (43) and RBF (44). Chronic administration raised creatinine clearance and reduced systolic blood pressure (45, 46). The Conrad group proposed the working model of the relaxin sustained-vasodilatory response, which develops in response to prolonged (hours or days) relaxin exposure (47). It was hypothesized that vascular endothelial growth factor (VEGF) initiates the gelatinase-ETB-receptor-NO-pathway in endothelium after relaxin activates the relaxin/insulin-like family (RXFP1) in vascular smooth muscles (47). Relaxin, therefore, stimulates the formation of endothelin through an alternative pathway with the help of matrix metalloproteinase gelatinases (45, 47-49), and endothelin in turn mediates vasodilation via nitric oxide (NO) synthesis. Increased NO levels in vascular beds (50), raised levels of plasma and urinary NO metabolites (51), as well as upregulated endothelial NO-synthase (NOS) in the arteries of pregnant rats (35, 52), and supported NO as a mediator of arterial vasodilation. Moreover, both the renal and systemic vasodilation response to pregnancy was prevented by NOS-inhibition (53); however, this occurred in association with significant hypertension, ie. potential model of preeclampsia. In pregnant rats, NOS-inhibition in a smaller dose - which did not induce hypertension - also reversed the systemic vasodilation and returned renal hemodynamics to nonpregnant levels. This supported a role for NO as a mediator of glomerular hyperfiltration and renal vasodilation in pregnancy (54). Relaxin binding sites are present in the brain (55) and intracerebroventricular administration of relaxin leads to activation of AVP containing neurons (56), increased thirst response (57), and AVP release (58). However, these studies were conducted only in animals (41, 59, 60) and results may differ significantly from human studies. In women undergoing in vitro fertilization (61, 62), and thus who lack relaxin, plasma osmolality nevertheless decreased, suggesting that primary vasodilation operates. Most relaxin-sensitive brain neurons are sensitive to ANG-II (63) and ANG-II (64, 65) stimulated relaxin-induced thirst. Thus, relaxin may be involved in the stimulation of thirst in pregnancy.

The vascular refractoriness to vasoconstrictors, including angiotensin II and norepinephrine, contributes to profound vasodilation in healthy pregnancy (66). Vascular insensitivity to ANG II during normal pregnancy may be the result of a decrease in ANG II receptors (67), or increased progesterone and prostacyclins (66), or relaxin’s effect (60). Notably, a similar refractoriness to the pressor effects of injected ANG II is exhibited by patients with cirrhosis and ascites, which is another state of vasodilation (68).

### 2.5. Implications for Preeclampsia

Understanding the physiology of healthy pregnancy will provide insights into the pathology of pregnancy, namely preeclampsia. This unique disorder of human pregnancy remains the leading cause of infant and maternal morbidity and mortality worldwide (69). A decrease in systemic and renal vascular resistance is the main physiologic adaptation of normal pregnancy, but this is not the case in preeclampsia. It is now clear that the hemodynamics of untreated preeclampsia is characterized by increased SVR (70, 71).

## 3. Results

Most likely, this loss of systemic vasodilation in preeclampsia is mediated by endothelial damage. Novel insights explain endothelial damage in preeclampsia by the release of antiangiogenic factors from an ischemic placenta. Increased placental production of soluble fms-like tyrosine kinase 1 (Flt1), an alternative splice variant of the VEGF receptor fms-like tyrosine kinase 1, might oppose physiologic vasorelaxation by blocking vasodilation induced by VEGF and placental growth factor (PGF) (72). A placenta-derived endoglin (Eng) - a soluble coreceptor for transforming growth factor (TGF)-β1 isoform - which is elevated in preeclamptic individuals, blocks TGF-β1-mediated activation of eNOS, and thus NO-dependant vasodilation (73).

Loss of vascular refractoriness to vasoconstrictors, which is characteristic of normal pregnancy (66), contributes to pronounced vasoconstriction in preeclampsia. In particular, Gant showed increased vascular sensitivity to ANG II in preeclampsia (74). However, the exact mechanism underlying this phenomenon is still unclear. Identified heterodimerization of AT1-receptors (receptors for ANG II) and B2-receptors (receptors for bradykinin) mediates, at least in part, an increased responsiveness to ANG II in preeclamptic hypertensive women (75). Stimulatory IgG autoantibodies against AT1 receptors found in preeclamptic women may also provoke this exaggerated pressor response to ANG II (76). Plasma ANG (1-7) which produces depressor, vasodilatory, and antihypertensive effects, is increased by 51% in normal pregnancy; in contrary, in preeclamptic subjects plasma ANG is decreased significantly and this might contribute to ANG II sensitivity as well (77).

## 4. Conclusions

Against the background of the Primary Arterial Vasodilation hypothesis, it is obvious that a reversal of systemic vasodilatation in pregnancy, without subsequent activation of the RAAS (78), will evoke a reversal of all links in the chain of events in normal pregnancy adaptation, therefore may cause preeclampsia. That is to say, a decrease in renal vasodilation will decrease GFR. A decrease in GFR will impair the aldosterone escape mechanism with a subsequent increase in sodium retention. Increased sodium retention will increase water retention and vascular sensitivity to vasoconstrictors, thus causing hypertension and edema. Increased ANG sensitivity through hemodynamic (79) and non-hemodynamic (80) mechanisms will enhance glomerular permeability to macromolecules and induce proteinuria. Thus pathological reversal of systemic vasorelaxation abrogates hemodynamic and homeostatic changes in pregnancy and leads to hypertension, edema and proteinuria, the diagnostic triad of preeclampsia.

## References

[1] Chapman AB, Abraham WT, Zamudio S, Coffin C, Merouani A, Young D (1998). Temporal relationships between hormonal and hemodynamic changes in early human pregnancy.. Kidney Int..

[2] van Buul EJ, Steegers EA, Jongsma HW, Eskes TK, Thomas CM, Hein PR (1995). Haematological and biochemical profile of uncomplicated pregnancy in nulliparous women; a longitudinal study.. Neth J Med..

[3] Hytten F (1985). Blood volume changes in normal pregnancy.. Clin Haematol..

[4] Lukaski HC, Siders WA, Nielsen EJ, Hall CB (1994). Total body water in pregnancy: assessment by using bioelectrical impedance.. Am J Clin Nutr..

[5] Wilson M, Morganti AA, Zervoudakis I, Letcher RL, Romney BM, Von Oeyon P (1980). Blood pressure, the renin-aldosterone system and sex steroids throughout normal pregnancy.. Am J Med..

[6] Sealey JE, Itskovitz-Eldor J, Rubattu S, James GD, August P, Thaler I (1994). Estradiol- and progesterone-related increases in the renin-aldosterone system: studies during ovarian stimulation and early pregnancy.. J Clin Endocrinol Metab..

[7] Paller MS, Gregorini G, Ferris TF (1989). Pressor responsiveness in pseudopregnant and pregnant rats: role of maternal factors.. Am J Physiol..

[8] Chapman AB, Zamudio S, Woodmansee W, Merouani A, Osorio F, Johnson A (1997). Systemic and renal hemodynamic changes in the luteal phase of the menstrual cycle mimic early pregnancy.. Am J Physiol..

[9] Robb AO, Mills NL, Din JN, Smith IB, Paterson F, Newby DE (2009). Influence of the menstrual cycle, pregnancy, and preeclampsia on arterial stiffness.. Hypertension..

[10] Balasch J, Arroyo V, Carmona F, Llach J, Jimenez W, Pare JC (1991). Severe ovarian hyperstimulation syndrome: role of peripheral vasodilation.. Fertil Steril..

[11] Hytten FE, Robertson EG (1971). Maternal water metabolism in pregnancy.. Proc R Soc Med..

[12] Newman RL (1957). Serum electrolytes in pregnancy, parturition, and puerperium.. Obstet Gynecol..

[13] Lindheimer MD, Davison JM (1995). Osmoregulation, the secretion of arginine vasopressin and its metabolism during pregnancy.. Eur J Endocrinol..

[14] Schrier RW, Briner VA (1991). Peripheral arterial vasodilation hypothesis of sodium and water retention in pregnancy: implications for pathogenesis of preeclampsia-eclampsia.. Obstet Gynecol..

[15] Schrier RW (1990). Body fluid volume regulation in health and disease: a unifying hypothesis.. Ann Intern Med..

[16] Robson SC, Hunter S, Boys RJ, Dunlop W (1989). Serial study of factors influencing changes in cardiac output during human pregnancy.. Am J Physiol..

[17] Schrier RW (1988). Pathogenesis of sodium and water retention in high-output and low-output cardiac failure, nephrotic syndrome, cirrhosis, and pregnancy (2).. N Engl J Med..

[18] August P, Lenz T, Ales KL, Druzin ML, Edersheim TG, Hutson JM (1990). Longitudinal study of the renin-angiotensin-aldosterone system in hypertensive pregnant women: deviations related to the development of superimposed preeclampsia.. Am J Obstet Gynecol..

[19] Davison JM (1984). Renal haemodynamics and volume homeostasis in pregnancy.. Scand J Clin Lab Invest Suppl..

[20] Phippard AF, Horvath JS, Glynn EM, Garner MG, Fletcher PJ, Duggin GG (1986). Circulatory adaptation to pregnancy--serial studies of haemodynamics, blood volume, renin and aldosterone in the baboon (Papio hamadryas).. J Hypertens..

[21] Gonzalez-Campoy JM, Romero JC, Knox FG (1989). Escape from the sodium-retaining effects of mineralocorticoids: role of ANF and intrarenal hormone systems.. Kidney Int..

[22] Knight S, Snellen H, Humphreys M, Baylis C (2007). Increased renal phosphodiesterase-5 activity mediates the blunted natriuretic response to ANP in the pregnant rat.. Am J Physiol Renal Physiol..

[23] Boron WF, Boulpaep EL (2005). Medical physiology..

[24] Dhanjal MK, Owen EP, Anthony JA, Davidson JS, Rayner BL (2006). Association of pre-eclampsia with the R563Q mutation of the beta-subunit of the epithelial sodium channel.. BJOG..

[25] Salas SP, Giacaman A, Vio CP (2004). Renal and hormonal effects of water deprivation in late-term pregnant rats.. Hypertension..

[26] Pike RL (1976). Soidum requirement of the rat during pregnancy.. Perspect Nephrol Hypertens..

[27] Salas SP, Marshall G, Gutierrez BL, Rosso P (2006). Time course of maternal plasma volume and hormonal changes in women with preeclampsia or fetal growth restriction.. Hypertension..

[28] Tamas P, Worgall S, Sulyok E, Rascher W (1994). Renal electrolyte and water handling in normal pregnancy: possible role of endothelin-1.. Eur J Obstet Gynecol Reprod Biol..

[29] Rosenbloom AA, Sack J, Fisher DA (1975). The circulating vasopressinase of pregnancy: species comparison with radioimmunoassay.. Am J Obstet Gynecol..

[30] Davison JM, Sheills EA, Barron WM, Robinson AG, Lindheimer MD (1989). Changes in the metabolic clearance of vasopressin and in plasma vasopressinase throughout human pregnancy.. J Clin Invest..

[31] Durr JA, Stamoutsos B, Lindheimer MD (1981). Osmoregulation during pregnancy in the rat. Evidence for resetting of the threshold for vasopressin secretion during gestation.. J Clin Invest..

[32] Davison JM, Shiells EA, Philips PR, Lindheimer MD (1988). Serial evaluation of vasopressin release and thirst in human pregnancy. Role of human chorionic gonadotrophin in the osmoregulatory changes of gestation.. J Clin Invest..

[33] Davison JM, Gilmore EA, Durr J, Robertson GL, Lindheimer MD (1984). Altered osmotic thresholds for vasopressin secretion and thirst in human pregnancy.. Am J Physiol..

[34] Ohara M, Martin PY, Xu DL, St John J, Pattison TA, Kim JK (1998). Upregulation of aquaporin 2 water channel expression in pregnant rats.. J Clin Invest..

[35] Xu DL, Martin PY, St John J, Tsai P, Summer SN, Ohara M (1996). Upregulation of endothelial and neuronal constitutive nitric oxide synthase in pregnant rats.. Am J Physiol..

[36] Fushimi K, Uchida S, Hara Y, Hirata Y, Marumo F, Sasaki S (1993). Cloning and expression of apical membrane water channel of rat kidney collecting tubule.. Nature..

[37] Asahina Y, Izumi N, Enomoto N, Sasaki S, Fushimi K, Marumo F (1995). Increased gene expression of water channel in cirrhotic rat kidneys.. Hepatology..

[38] Xu DL, Martin PY, Ohara M, St John J, Pattison T, Meng X (1997). Upregulation of aquaporin-2 water channel expression in chronic heart failure rat.. J Clin Invest..

[39] Buemi M, D'Anna R, Di Pasquale G, Floccari F, Ruello A, Aloisi C (2001). Urinary excretion of aquaporin-2 water channel during pregnancy.. Cell Physiol Biochem..

[40] Conrad KP, Debrah DO, Novak J, Danielson LA, Shroff SG (2004). Relaxin modifies systemic arterial resistance and compliance in conscious, nonpregnant rats.. Endocrinology..

[41] Debrah DO, Novak J, Matthews JE, Ramirez RJ, Shroff SG, Conrad KP (2006). Relaxin is essential for systemic vasodilation and increased global arterial compliance during early pregnancy in conscious rats.. Endocrinology..

[42] Danielson LA, Kercher LJ, Conrad KP (2000). Impact of gender and endothelin on renal vasodilation and hyperfiltration induced by relaxin in conscious rats.. Am J Physiol Regul Integr Comp Physiol..

[43] Dschietzig T, Teichman S, Unemori E, Wood S, Boehmer J, Richter C (2009). Intravenous recombinant human relaxin in compensated heart failure: a safety, tolerability, and pharmacodynamic trial.. J Card Fail..

[44] Smith MC, Danielson LA, Conrad KP, Davison JM (2006). Influence of recombinant human relaxin on renal hemodynamics in healthy volunteers.. J Am Soc Nephrol..

[45] Palejwala S, Stein DE, Weiss G, Monia BP, Tortoriello D, Goldsmith LT (2001). Relaxin positively regulates matrix metalloproteinase expression in human lower uterine segment fibroblasts using a tyrosine kinase signaling pathway.. Endocrinology..

[46] Teichman SL, Unemori E, Dschietzig T, Conrad K, Voors AA, Teerlink JR (2009). Relaxin, a pleiotropic vasodilator for the treatment of heart failure.. Heart Fail Rev..

[47] McGuane JT, Danielson LA, Debrah JE, Rubin JP, Novak J, Conrad KP (2011). Angiogenic growth factors are new and essential players in the sustained relaxin vasodilatory pathway in rodents and humans.. Hypertension..

[48] Fernandez-Patron C, Radomski MW, Davidge ST (1999). Vascular matrix metalloproteinase-2 cleaves big endothelin-1 yielding a novel vasoconstrictor.. Circ Res..

[49] Jeyabalan A, Novak J, Danielson LA, Kerchner LJ, Opett SL, Conrad KP (2003). Essential role for vascular gelatinase activity in relaxin-induced renal vasodilation, hyperfiltration, and reduced myogenic reactivity of small arteries.. Circ Res..

[50] Weiner C, Martinez E, Zhu LK, Ghodsi A, Chestnut D (1989). In vitro release of endothelium-derived relaxing factor by acetylcholine is increased during the guinea pig pregnancy.. Am J Obstet Gynecol..

[51] Conrad KP, Joffe GM, Kruszyna H, Kruszyna R, Rochelle LG, Smith RP (1993). Identification of increased nitric oxide biosynthesis during pregnancy in rats.. FASEB J..

[52] Weiner CP, Knowles RG, Moncada S (1994). Induction of nitric oxide synthases early in pregnancy.. Am J Obstet Gynecol..

[53] Kassab S, Miller MT, Hester R, Novak J, Granger JP (1998). Systemic hemodynamics and regional blood flow during chronic nitric oxide synthesis inhibition in pregnant rats.. Hypertension..

[54] Cadnapaphornchai MA, Ohara M, Morris KG, Jr., Knotek M, Rogachev B, Ladtkow T (2001). Chronic NOS inhibition reverses systemic vasodilation and glomerular hyperfiltration in pregnancy.. Am J Physiol Renal Physiol..

[55] Osheroff PL, Phillips HS (1991). Autoradiographic localization of relaxin binding sites in rat brain.. Proc Natl Acad Sci U S A..

[56] McKinley MJ, Colvill LM, Giles ME, Oldfield BJ (1997). Distribution of Fos-immunoreactivity in rat brain following a dipsogenic dose of captopril and effects of angiotensin receptor blockade.. Brain Res..

[57] Summerlee AJ, Robertson GF (1995). Central administration of porcine relaxin stimulates drinking behaviour in rats: an effect mediated by central angiotensin II.. Endocrine..

[58] Mumford AD, Parry LJ, Summerlee AJ (1989). Lesion of the subfornical organ affects the haemotensive response to centrally administered relaxin in anaesthetized rats.. J Endocrinol..

[59] Weisinger RS, Burns P, Eddie LW, Wintour EM (1993). Relaxin alters the plasma osmolality-arginine vasopressin relationship in the rat.. J Endocrinol..

[60] Danielson LA, Sherwood OD, Conrad KP (1999). Relaxin is a potent renal vasodilator in conscious rats.. J Clin Invest..

[61] Smith MC, Murdoch AP, Danielson LA, Conrad KP, Davison JM (2006). Relaxin has a role in establishing a renal response in pregnancy.. Fertil Steril..

[62] Johnson MR, Brooks AA, Steer PJ (1996). The role of relaxin in the pregnancy associated reduction in plasma osmolality.. Hum Reprod..

[63] Sunn N, Egli M, Burazin TC, Burns P, Colvill L, Davern P (2002). Circulating relaxin acts on subfornical organ neurons to stimulate water drinking in the rat.. Proc Natl Acad Sci U S A..

[64] Sinnayah P, Burns P, Wade JD, Weisinger RS, McKinley MJ (1999). Water drinking in rats resulting from intravenous relaxin and its modification by other dipsogenic factors.. Endocrinology..

[65] Geddes BJ, Parry LJ, Summerlee AJ (1994). Brain angiotensin-II partially mediates the effects of relaxin on vasopressin and oxytocin release in anesthetized rats.. Endocrinology..

[66] Gant NF, Worley RJ, Everett RB, MacDonald PC (1980). Control of vascular responsiveness during human pregnancy.. Kidney Int..

[67] Baker PN, Broughton Pipkin F, Symonds EM (1990). Platelet angiotensin II binding and plasma renin concentration, plasma renin substrate and plasma angiotensin II in human pregnancy.. Clin Sci (Lond)..

[68] LARAGH JH (1962). Hormones and the Pathogenesis of Congestive Heart Failure:Vasopressin, Aldosterone, and Angiotensin II: Further Evidence for Renal-Adrenal Interaction from Studies in Hypertension and in Cirrhosis.. Circulation..

[69] Duley L (2009). The global impact of pre-eclampsia and eclampsia.. Semin Perinatol..

[70] Dennis AT, Castro J, Carr C, Simmons S, Permezel M, Royse C (2012). Haemodynamics in women with untreated pre-eclampsia.. Anaesthesia..

[71] Visser W, Wallenburg HC (1991). Central hemodynamic observations in untreated preeclamptic patients.. Hypertension..

[72] Maynard SE, Min JY, Merchan J, Lim KH, Li J, Mondal S (2003). Excess placental soluble fms-like tyrosine kinase 1 (sFlt1) may contribute to endothelial dysfunction, hypertension, and proteinuria in preeclampsia.. J Clin Invest..

[73] Venkatesha S, Toporsian M, Lam C, Hanai J, Mammoto T, Kim YM (2006). Soluble endoglin contributes to the pathogenesis of preeclampsia.. Nat Med..

[74] Gant NF, Daley GL, Chand S, Whalley PJ, MacDonald PC (1973). A study of angiotensin II pressor response throughout primigravid pregnancy.. J Clin Invest..

[75] AbdAlla S, Lother H, el Massiery A, Quitterer U (2001). Increased AT(1) receptor heterodimers in preeclampsia mediate enhanced angiotensin II responsiveness.. Nat Med..

[76] Wallukat G, Homuth V, Fischer T, Lindschau C, Horstkamp B, Jupner A (1999). Patients with preeclampsia develop agonistic autoantibodies against the angiotensin AT1 receptor.. J Clin Invest..

[77] Merrill DC, Karoly M, Chen K, Ferrario CM, Brosnihan KB (2002). Angiotensin-(1-7) in normal and preeclamptic pregnancy.. Endocrine..

[78] Irani RA, Xia Y (2011). Renin angiotensin signaling in normal pregnancy and preeclampsia.. Semin Nephrol..

[79] Bohrer MP, Deen WM, Robertson CR, Brenner BM (1977). Mechanism of angiotensin II-induced proteinuria in the rat.. Am J Physiol..

[80] Axelsson J, Rippe A, Oberg CM, Rippe B (2012). Rapid, dynamic changes in glomerular permeability to macromolecules during systemic angiotensin II (ANG II) infusion in rats.. Am J Physiol Renal Physiol..

